# Estimation of the respiratory rate from ballistocardiograms using the Hilbert transform

**DOI:** 10.1186/s12938-022-01024-4

**Published:** 2022-08-04

**Authors:** Onno Linschmann, Steffen Leonhardt, Antti Vehkaoja, Christoph Hoog Antink

**Affiliations:** 1 Medical Information Technology, Helmholtz Institute, RWTH Aachen University, Aachen, Germany; 2 Faculty of Medicine and Health Technology, Tampere University, Tampere, Finland; 3 KIS*MED (AI Systems in Medicine), TU Darmstadt, Darmstadt, Germany

**Keywords:** Instantaneous breathing frequency, Respiration, Ballistocardiography

## Abstract

**Background:**

Measuring the respiratory rate is usually associated with discomfort for the patient due to contact sensors or a high time demand for healthcare personnel manually counting it.

**Methods:**

In this paper, two methods for the continuous extraction of the respiratory rate from unobtrusive ballistocardiography signals are introduced. The Hilbert transform is used to generate an amplitude-invariant phase signal in-line with the respiratory rate. The respiratory rate can then be estimated, first, by using a simple peak detection, and second, by differentiation.

**Results:**

By analysis of a sleep laboratory data set consisting of nine records of healthy individuals lasting more than 63 h and including more than 59,000 breaths, a mean absolute error of as low as 0.7 BPM for both methods was achieved.

**Conclusion:**

The results encourage further assessment for hospitalised patients and for home-care applications especially with patients suffering from diseases of the respiratory system like COPD or sleep apnoea.

## Background

In medical signals for obtaining the heart activity, such as electrocardiography (ECG), photoplethysmography (PPG) and ballistocardiography (BCG), respiration components with different strengths are present. While there are many approaches for extracting the respiration from ECG (ECG-derived respiration) and PPG, respiration extraction from BCG signals is still a neglected topic [1] even though in BCG signals the respiration component is usually the dominant one. In the last years, the importance of measuring the respiratory rate in a hospital setting has been reported in many publications, e.g. for the early prediction of deterioration [2, 3] or opioid-induced respiration depression with respect to postsurgery analgesia [4, 5]. As pointed out in [6], it is still one of the vital signs which is highly underestimated by medical staff who usually need to manually count it. Apart from a hospital setting, the respiratory rate is also an important measure in other settings including for example sleep analysis with respect to sleep-related breathing disorders, such as sleep apnoea [7]. For sleep analysis, polysomnography is the gold standard, but comes with many obtrusive sensors which may cause discomfort for the patients. This then leads to the “First Night Effect” and is therefore thought to be one of the main causes for it [8, 9]. BCG sensors can address these problems by providing a way of continuous, unobtrusive measurement of the respiratory rate [10] and in the case of polysomnography might even be able to replace most of the device [7, 11, 12]. However, it should be noted that in contrast to, e.g. wrist-worn devices for pulse oximetry and photoplethysmography (PPG), no information about the oxygen saturation can be obtained. Nevertheless, BCG sensors can detect changes in the morphological structure of the heart beat and thus provide valuable information [13].

Several approaches for extracting the respiratory rate from classical and unobtrusive signals exist. Analysis methods are usually based on either Fourier analysis, wavelet decomposition, or selective filtering combined with a peak detection. For example, Karlen et al. [14] as well as Watanabe et al. [15] developed algorithms based on the short-time Fourier analysis. The signal, either respiratory flow or a BCG signal, is first band-pass filtered to remove noise and in the case of the BCG signal also the modulated heart-related signal. The signal is then windowed and for each window the maximum frequency of the Fourier transformation is calculated. This frequency is assigned as the respiratory rate for that window. Zhu et al. [16] developed a method in which the BCG signal is decomposed by means of a wavelet decomposition. The respiratory rate is then obtained by a peak detection on one of the scales. Alihanka et al. [17] as well as Erkinjuntti et al. [18] band-pass filtered the BCG signal and applied a peak detection on the filtered signal to calculate the inter-breath intervals and thus the respiratory rate. Paalasmaa et al. [19] developed a filter-bank such that the signal is low-pass filtered at specific corner frequencies. A peak detection is applied on each of the low-pass filtered signals and the respiratory rate is chosen for a pre-defined window for the signal in which the inter-breath intervals exhibit the least variability. Their algorithm also includes a motion artefact detection such that segments with motion are discarded. Wang et al. [20] developed a method based on adaptive interference cancellation. Here, the respiration waveform was recovered using a BCG without respiration as a reference signal such that the heart-related signal and noise are adaptively filtered. Finally, Harada et al. [21] developed a peak detection with motion artefact suppression. First, the signal is low-pass filtered. Then, the inter-breath intervals are calculated. Inter-breath intervals are only accepted if the amplitudes of the signal surpass a certain threshold. Changes in posture are also tracked.

Different from the aforementioned methods, in this paper two new methods based on selective filtering and the Hilbert transform for obtaining the respiration from BCG signals are presented. Deviating from other approaches using the Hilbert transform for extracting the heart rate, as in, e.g. [22, 23], the phase signal of the Hilbert transform is used instead of the amplitude signal. The approach is similar to the one used in Empirical Mode Decomposition for estimating the instantaneous frequency in multi-component signals which was first postulated by Huang et al. [24]. A sleep laboratory data set consisting of nine records of healthy individuals lasting more than 63 h and including more than 59,000 breaths is used for assessing these approaches [25]. Furthermore, breathing rate variability (BRV) metrics [26] are used to assess whether short-term effects can be captured. The methods are compared with two methods from literature, i.e. from Karlen et al. and Paalasmaa et al. [14, 19].

The paper is structured as follows. First, breathing rate variability metrics are revised. Second, the results of our new approaches for estimating the respiratory rate in terms of mean absolute error, root-mean-square error and BRV parameters are presented, followed by a discussion and conclusion. Finally, the method, i.e. the new approaches for the extraction of the respiratory rate are introduced.

### Breathing rate variability

Analogous to the heart rate variability (HRV) metrics [27–29], breathing rate variability (BRV) metrics can be defined to capture statistical change in the inter-breath intervals (IBI) for resting individuals and thus the breathing rate variability [26, 30]. In [26] it was shown that several parameters in the time domain as well as in the frequency domain and the non-linear domains exhibit higher values in meditating individuals. It was speculated that breathing rate variability can capture the short-term effects of the nervous system and can be used to measure stress. In this paper, the following metrics are used for accessing the presented algorithms: mean inter-breath interval (MIBI), standard deviation of IBI intervals (SDBB) and root mean square of successive IBI differences (RMSSD). Additionally, very-low-frequency (VLF), low-frequency (LF) and high-frequency (HF) band measures such as VLF absolute power, LF absolute and relative power and HF absolute power have been compared.

## Results

The proposed approaches (see Methods section) were tested on a data set providing a full polysomnography and an additional BCG from nine healthy subjects in a sleep laboratory while sleeping [25]. The BCG sensor used in the study was an electro-mechanical film sensor (EMFi; Emfit Ltd, Vaajakoski, Finland). EMFI is an oriented polypropylene film which is placed between two electrodes. Pressure on the EMFI leads to an electric field by moving charges on the boundaries between air voids and the layered polypropylene. These charges produce mirror charges on the electrodes which can thus be measured [31]. The sensor (${30}\,\hbox {cm} \times {60}\,\hbox {cm}$) was positioned under a thin foam layer on top of a mattress. The BCG signal was sampled with ${200}\,\hbox {Hz}$. A respiratory flow signal from the polysomnography was used as reference (sampling rate of ${10}\,\hbox {Hz}$) for extracting the ground truth for the respiratory rate. The respiratory rate was extracted using peak detection. For that, the signal was filtered by a second-order Butterworth bandpass filter with cut-off frequencies at ${0.1}\,\hbox {Hz}$ and ${0.5}\,\hbox {Hz}$. Then, peaks were detected using the *findpeaks*-function of MATLAB (The Mathworks). The minimum peak distance was defined to be 2.2 s to avoid the annotation of local maxima. Since artefacts also occurred in the reference signal, each reference signal and its peak detection were manually checked and artefacts annotated to exclude them in the evaluation. For each subject, the respiratory rate was calculated using the previously presented methods. First, a peak detection on the phase signal of the Hilbert transform, and second, the filtered derivative of the Hilbert transform’s phase signal were calculated. For the peak detection, the same parameters as for the reference were used. The performance of these algorithms was compared with the approach from Karlen et al. [14] and with the algorithm of Paalasmaa et al. [19] by means of the mean absolute error (MAE) and the root-mean-square error (RMSE). As visible in Table 1, both methods are able to accurately estimate the respiratory rate. While the performance of the peak detection achieves MAEs of as small as ${0.69}\,\hbox {BPM}$ (mean across all individuals is ${1.19}\,\hbox {BPM}$), the filtered Hilbert phase derivative estimation is slightly less accurate with MAEs as small as ${0.71}\,\hbox {BPM}$ (mean across all individuals is ${1.49}\,\hbox {BPM}$). As visible through the RMSEs, the trajectory and thus breathing rate variability can also be captured. Another observation that can be made is that for individuals 5 and 6, the estimation by the filtered Hilbert phase derivative is much worse in comparison to the peak detection. In these recordings, strong motion artefacts were present. The peak detection has the advantage that the next peak location is limited to a physiological meaningful interval and therefore is naturally closer to the real value. In contrast, the frequency range of $f_{RR,\text {filt.}}$ is not limited and no motion artefact detection and exclusion criterion were used. As visible in Table 2, the approach from [14] and the algorithm from [19] are less accurate for all subjects even though no rejection of motion artefacts as in the proposed algorithms from Paalasmaa et al. was used. It can also be seen that again the estimations of subjects 5 and 6 are less accurate. The newly presented algorithms also outperform these approaches in terms of RMSE.

**Table 1:** MAE and RMSE for the two newly developed algorithms, namely the peak detection and filtered phase derivative for each individual data set, as well as mean and variance across the data sets

Subject	$\text {MAE}_\text {peak}$ (BPM)	$\text {MAE}(f_{RR,\text {filt.}})$ (BPM)	$\text {RMSE}_\text {peak}$ (BPM)	$\text {RMSE}(f_{RR,\text {filt.}})$ (BPM)
1	1.0133	0.9338	2.4795	2.1606
2	1.0378	1.0966	2.2614	2.7046
3	1.2212	1.2146	2.8401	2.8260
4	0.8632	0.7854	1.9597	1.9611
5	2.0258	2.9025	3.5064	5.0844
6	1.8381	3.5504	3.2011	6.0620
7	0.7474	0.8357	1.6673	2.0976
8	1.2305	1.3888	2.5367	2.8137
9	0.6940	0.7077	1.8490	1.8645
Mean	1.1857	1.4906	2.4779	3.0638
Variance	0.2155	1.0409	0.3854	2.2155

**Table 2:** MAE and RMSE of reference methods of [14] and [19] from literature

Subject	$\text {MAE}_{[14]}$ (BPM)	$\text {MAE}_{[19]}$ (BPM)	$\text {RMSE}_{[14]}$ (BPM)	$\text {RMSE}_{[19]}$ (BPM)
1	2.3609	2.2394	3.0778	3.5719
2	2.7337	2.8780	3.6254	4.4147
3	3.0300	3.0364	4.0274	4.6449
4	2.3358	2.1109	3.0538	3.5097
5	3.8015	4.7098	5.2576	8.3815
6	4.2238	4.4550	6.2964	6.8651
7	2.2534	2.4365	2.9410	4.1007
8	2.3549	2.7381	3.0796	4.4749
9	2.4976	2.5958	3.2010	3.9580
Mean	2.8435	3.0222	3.84	4.882
Variance	0.5084	0.8714	1.3898	2.7104

**Fig. 1:**
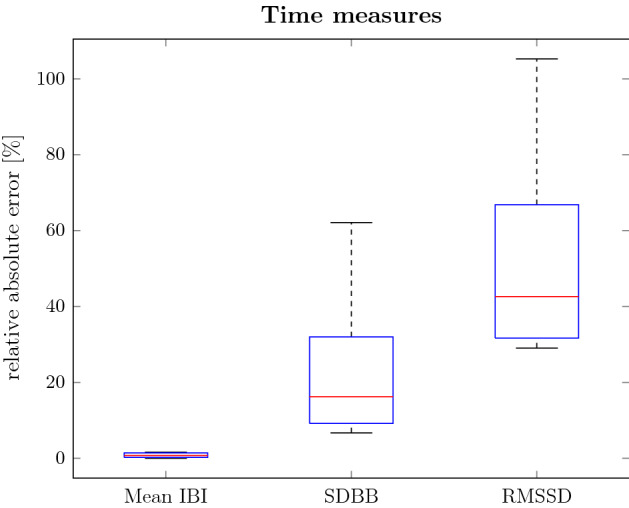
Relative absolute error of time-domain measures for all subjects

**Fig. 2:**
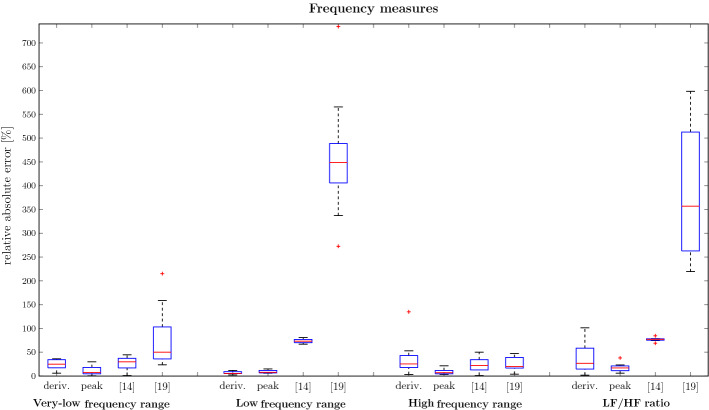
Relative absolute error of frequency-domain measures for all subjects and all methods

To further evaluate whether BRV can be captured, breathing rate variability parameters for each method and the reference were compared. Note that BRV parameters based on the change in respiration intervals cannot be reflected by $f_{RR,\text {filt.}}$ as it is a continuous function and does not provide inter-breath intervals. This also holds for the baseline methods. The BRV time measures mean IBI, SDBB and RMSSD, and the frequency measures VLF, absolute and relative LF power, absolute and relative HF power and LF–HF ratio were evaluated. From Fig. 1, it can be seen that the mean IBI across all records can be captured with a vanishing error. In contrast, it can also be seen that local dynamics reflected by SDBB and RMSSD are only captured with a median error of around ${16}\,{\%}$ and ${42}\,{\%}$, respectively. In terms of frequency parameters, it can be seen in Fig. 2 that the peak detection captures the power in each frequency band with a median error between ${7}\,{\%}$ and ${17}\,{\%}$. The filtered phase derivative’s median error lies between ${5}\,{\%}$ and ${25}\,{\%}$ outperforming the peak detection only in the LF band. It is visible that the HF band has a particularly high error and large variance, which could be caused by insufficient filtering such that there are residual BCG shape-related artefacts. This supports the findings from the SDBB and RSSD, i.e. long-term trends are captured well while local features lack accuracy. Compared with the baseline methods, it can be stated that both methods capture the VLF and LF range more accurately and are similarly accurate in the HF range. It should be noted that for both baseline methods, different sensor setups were used and especially for the method of [19] slight changes in waveform can have a strong impact diminishing the accuracy which is especially visible in the lower frequency measures.

## Discussion and conclusion

Two methods based on the Hilbert transform and the Hilbert–Huang spectrum were presented to accurately and robustly estimate the respiratory rate from BCG data. It was shown that the methods are invariant to changing amplitudes due to a change in mechanical coupling between the sensor and the patient. It could also be shown that, while the error in mean respiratory rate is low, higher order statistics reflected by BRV parameters still lack accuracy, especially in the case of the filtered phase derivative. The estimation is computationally inexpensive since the Hilbert transform can be computed by means of the fast Fourier transform. Nonetheless, for employing the estimation in real-time, a windowed approach would be necessary. Nevertheless, the higher accuracy outweighs the increased computational cost. However, the analysis also has some limitations. First, for evaluation of the algorithms only healthy subjects were investigated. Therefore, a proper analysis with regard to different sleep-related diseases has to be conducted. In terms of COPD patients and patients suffering from sleep apnoea, it can be expected that the methods, especially the phase derivate can still be used. In COPD patients, a change in waveform and amplitude can be expected which does not severely influence the methods since they are chosen in a way that waveform and periodicity are separated. Since the periodic waveform is disrupted during apnoea phases, two effects can be expected. First, the estimated respiratory rate by the first approach will drop. Second, there might still be peaks in the phase signal during these phases such that the second approach might detect them. The amplitude signal might be employed to tackle this problem. Last, the algorithms were compared on our data set. Therefore, an influence of the data quality cannot completely be ruled out. However, the compared methods only provide a much coarser resolution and average the respiratory rate and thus are less accurate and are therefore less suitable for BRV calculations. For future work, a fusion strategy using several unobtrusive sensors could improve the estimation as well as different filtering procedures and motion artefact detection. Especially, a comparison and combination with wrist-worn sensors would be interesting. Furthermore, BRV parameters based on the instantaneous respiratory rate generated by the proposed approach should be defined and evaluated.

## Method

Due to the positioning of the sensing elements of cardiac measurement modalities (for example ECG and BCG), the recorded signals do not only capture the periodic reaction of the heart, but also capture respiration in form of a frequency modulation and/or amplitude modulation. Thus, they can be described by so-called “intrinsic mode functions” (IMF). An IMF is charaterised by a cosine function with a time-dependent frequency and amplitude. The BCG signal can be assumed to consist of mainly two intrinsic mode functions [22, 23], in form of an amplitude modulation, i.e. an IMF associated with the heart rate superposed by one associated with the respiratory rate, i.e.

1 $$\begin{aligned} s(t) = \sum _{i\in \{\text {HR,RR}\}} A_\text {i}(t) \cdot \text {cos}(2\pi f_\text {i}t) + n(t) \text {.} \end{aligned}$$

Here, *A* describes the amplitude signal, *f* describes the frequency and *n*(*t*) describes additive noise and motion artefacts. HR and RR refer to the IMF for the heart rate (HR) and the respiratory rate (RR). Similar to [22, 23], to separate the respiratory IMF from the one associated with the heart, a Butterworth bandpass filter with cut-off frequencies at ${0.1}\,\hbox {Hz}$ and ${0.5}\,\hbox {Hz}$, according to respiration rates of 6 and 30 breaths per minute (BPM), can be used. The residual signal can thus be assumed to consist only of the IMF for the respiration as well as additive filtered noise and motion artefacts

2 $$\begin{aligned} \tilde{s}(t) = A_\text {RR}(t) \cdot \text {cos}(2\pi f_\text {RR}(t)t) + \tilde{n}(t)\text {.} \end{aligned}$$

Applying the Hilbert transform to this IMF then leads to the so-called analytic component

3 $$\begin{aligned} \tilde{s}_+(t) \approx A_\text {RR}(t) \cdot \text {exp}(i2\pi f_\text {RR}(t)t)\text {,} \end{aligned}$$

where *i* denotes the imaginary unit. The analytic component is a complex phasor, where $A_\text {RR}(t)$ represents the waveform of the signal and the complex exponential function its periodic repetition. Therefore, the phase signal of $\tilde{s}_+$ can be assumed to be periodic with the respiratory rate. The respiratory rate can then be extracted by differentiation of the phase signal $\varphi _{\tilde{\mathrm{s}}_+}(t)$:

4 $$\begin{aligned} f_\text {RR}(t)&= \frac{1}{2\pi } \frac{d\varphi _{\tilde{\mathrm{s}}_+}(t)}{dt} \quad \text {with} \end{aligned}$$

5 $$\begin{aligned} \varphi _{\tilde{\mathrm{s}}_+}(t)&= \text {arg}(\tilde{s}_+)\text {.} \end{aligned}$$

**Fig. 3:**
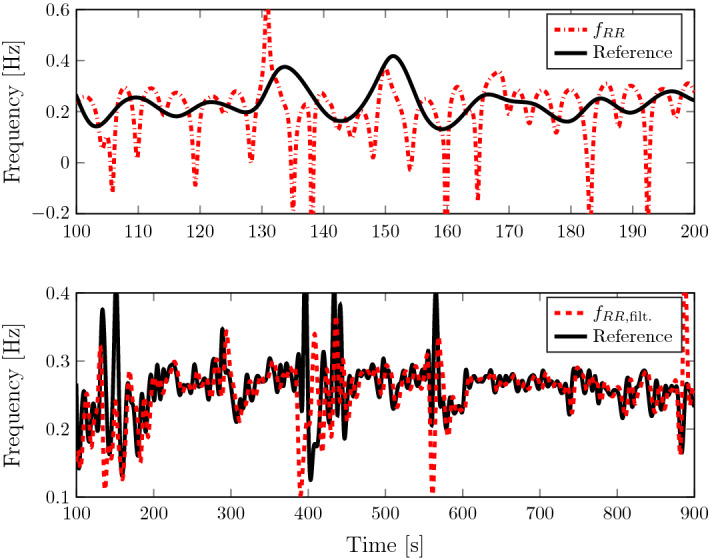
Frequency extraction using the derivative of the phase signal and its filtered version. The black solid line is the reference respiratory rate signal

This frequency signal is further median-filtered to remove artefacts due to phase discontinuities which appear periodically with the respiratory rate. Additionally, the signal is low-pass filtered to remove BCG shape-related artefacts [19] in which additional peaks in the phase signal of one respiration appear (see Fig. 3). For filtering, a Butterworth low-pass filter with a cut-off frequency of ${0.1}\,\hbox {Hz}$ is applied since the oscillation’s frequency was found to be around ${0.2}\,\hbox {Hz}$.

**Fig. 4:**
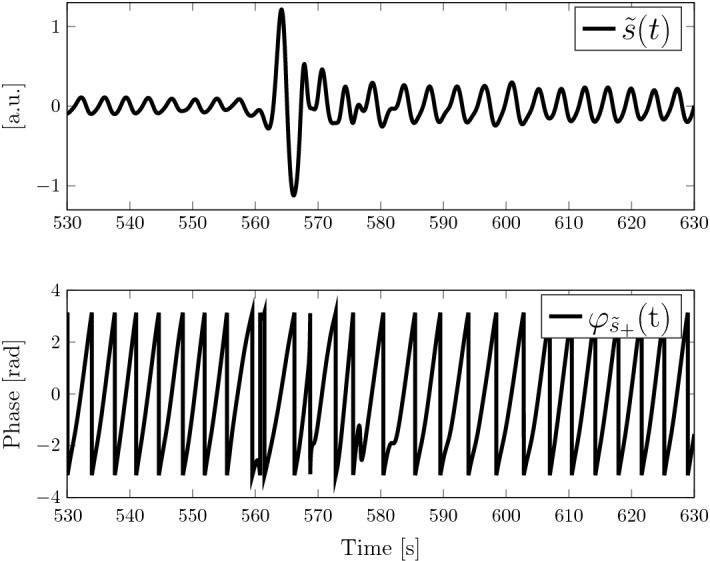
Top: filtered BCG signal. Bottom: phase signal of Hilbert transform. At around ${560}\,\hbox {s}$, a motion artefact occurs which follows a change in signal level. The phase signal is independent of that change

**Fig. 5:**
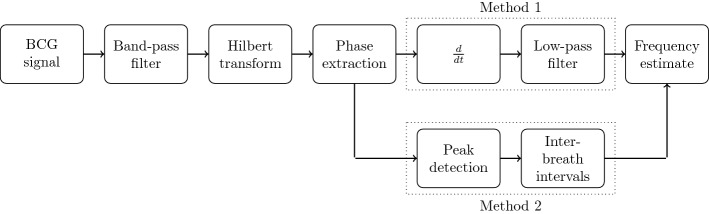
Workflow of the proposed algorithms

Furthermore, the frequency can also be calculated with a peak detection directly on the phase signal $\varphi _{\tilde{s}_+}$ since it has clear peaks at its discontinuities, i.e. at phase jumps from $\pi$ to $-\pi$. An example can be seen in the bottom plot of Fig. 4. Each of the saws represents one breathing cycle. For the peak detection, it is advantageous that the phase signal is independent of amplitude changes in the BCG signal which often appear due to a shift in position of the subject on the sensor (see Fig. 4). The complete workflow is depicted in Fig. 5.

As a baseline for comparison, two methods from literature were chosen. First, the approach from Karlen et al. [14] originally for flow signals was adapted to BCG signals. The BCG signal was resampled to ${50}\,\hbox {Hz}$ and band-pass filtered with a third-order Butterworth bandpass filter with cut-off frequencies at ${0.1}\,\hbox {Hz}$ and ${0.5}\,\hbox {Hz}$ to remove the heart’s IMF. Subsequently, the signal was windowed by a Hamming window. Each segment has a length of 2048 samples which corresponds to ${40.96}\,\hbox {s}$. The signal was then transformed into the Fourier domain. The DC-component was removed and only the frequencies smaller than ${8}\,\hbox {Hz}$ are analysed. The peak in the spectrum is chosen to be the respiratory rate. Second, the method from Paalasmaa et al. [19] was used. The BCG signal here was resampled to ${300}\,\hbox {Hz}$. To discard segments in which motion artefacts occur, the signal was split into segments of ${10}\,\hbox {s}$. Each window’s peak-to-peak value, i.e. lowest to highest amplitude, was calculated. If the peak-to-peak value was found to be larger than twice the average, 15 s before and after the segment were discarded. The signal was then low-pass filtered with four low-pass filters with different cut-off frequencies, i.e. ${0.154}\,\hbox {Hz}$, ${0.22}\,\hbox {Hz}$, ${0.33}\,\hbox {Hz}$ and ${0.5}\,\hbox {Hz}$. A peak detection was applied on all four signals. For every 3 s, a respiratory rate was chosen from the four signals by the inter-breath intervals. The rate of that signal was chosen in which the variability with respect to the amplitude of the last five cycles was smallest. The variability *V* for each interval was calculated by

6 $$\begin{aligned}&V = \max _{2 \le i \le 5} = |\text {log}(b_i)-\text {log}(b_{i-1})|, \end{aligned}$$

with $b_i$ being the amplitudes at the start of each interval.

## Data Availability

Not applicable.

## Abbreviations

BCG: Ballistocardiography
BPM: Breaths per minute
BRV: Breathing rate variability
ECG: Electrocardiography
EMFi: Electromechanical film
HF: High frequency
HR: Heart rate
IBI: Inter-breath interval
IMF: Intrinsic mode function
LF: Low frequency
MAE: Mean absolute error
MIBI: Mean inter-breath interval
PPG: Photoplethysmography
SDBB: Standard deviation of IBI
RMSE: Root-mean-squared error
RMSSD: Root-mean-squared value of IBI differences
RR: Respiratory rate
VLF: Very low frequency
